# A Combined α-Synuclein/Fibril (SynFib) Model of Parkinson-Like Synucleinopathy Targeting the Nigrostriatal Dopamine System

**DOI:** 10.3233/JPD-223452

**Published:** 2022-12-16

**Authors:** Anders Björklund, Fredrik Nilsson, Bengt Mattsson, Deirdre B. Hoban, Malin Parmar

**Affiliations:** Developmental and Regenerative Neurobiology, Department of Experimental Medical Science, Wallenberg Neuroscience Center, Lund University, Lund, Sweden

**Keywords:** adeno-associated virus, disease modeling, inflammation, motor behavior, mouse, Parkinson’s disease, pre-formed fibrils, rat

## Abstract

Injections of pre-formed α-synuclein fibrils (PFFs) or overexpression of α-synuclein using AAV vectors are commonly used as models of Parkinson-like synucleinopathy in rats and mice. In the modified method reviewed here, the “*SynFib*” model, the PFFs and the AAV vector are administered together unilaterally into the substantia nigra. This approach combines the key features of these two models, i.e., the generation of toxic α-synuclein aggregates and Lewy body-like inclusions, in combination with the increased vulnerability caused by increased cellular levels of α-synuclein. The combined AAV/PFF delivery offers several advantages over the standard PFF model due to the enhanced and accelerated α-synuclein pathology and microglial response induced by the PFF seeds in the presence of an elevated α-synuclein level. Injection of the AAV/PFF mixture into the substantia nigra makes it possible to target a larger proportion of the nigral dopamine neurons and obtain a level of dopamine cell loss (>60%) needed to induce significant impairments in drug-induced and spontaneous motor tests. The SynFib model shares attractive features of the standard 6-OHDA lesion model: a single unilateral stereotaxic intervention; pathology and cell loss developing over a short time span; and the possibility to monitor the degenerative changes using tests of motor behavior.

For decades, the 6-hydroxydopamine (6-OHDA) lesion model, applied unilaterally in rats and mice, has been a standard experimental tool in Parkinson’s disease (PD) research. This toxin-based model was introduced in the 1960 s [1] to induce damage to the nigrostriatal dopamine (DA) system akin to the DA neuron loss seen in PD patients. The 6-OHDA model is attractive and useful due to the robust and easily quantifiable motor deficits which correlate with the extent of DA neuron loss [2]. The major shortcoming of this model, however, is that it does not reproduce any of the pathological features related to the progressive synucleinopathy that is characteristic of the human disease.

The discovery of the role of α-synuclein (α-syn) in the pathogenesis of PD has presented an alternative approach to PD disease modeling based on either forced expression of wild-type or mutated α-syn using transgenic techniques, viral vector mediated transfer of α-syn, or local injection of pathogenic pre-formed α-syn fibrils (PFFs) [3, 4]. The adeno-associated virus (AAV)-α-syn and PFF models have been adapted specifically for studies of synucleinopathy using stereotaxic delivery into either substantia nigra (SN) or striatum. These models have proven highly useful, but have clear limitations as routine experimental tools:

*In the AAV-* α*-syn model*, the degenerative changes develop slowly and substantial DA neuron cell loss is obtained only with very high expression levels of the protein, corresponding to at least 4-5-fold above normal [5–7], i.e., at levels of α-syn that far exceed those seen in human PD. Moreover, the microglial/inflammatory response which is a characteristic feature of the human disease is transient and usually of modest magnitude.

*In the PFF model* the PFFs, injected either in striatum [8–10] or SN [11–13], act as seeds for the formation of α-syn aggregates, leading to a slowly developing Lewy body like pathology that is observed not only in nigral DA neurons but also widespread in other brain regions, such as amygdala and cortex [10, 12], due to retrograde transport in neuron projecting to the injected area. DA cell loss develops very slowly and is observed only after 5-6 months. The PFF model has been widely used in mice but appears less useful in rats where the cell loss is more variable in magnitude, in the order of 20–50% at 4–6 months post-injection, and not sufficient to induce robust impairments in standard motor tests [9, 12, 13]. As in the AAV-α-syn model the microglial/inflammatory response is transient and rather modest in magnitude [13, 14].

The idea of combining the two approaches–AAV mediated α-syn delivery with the addition of PFF seeds–is based on the finding that the generation, magnitude, and speed of progression of toxic α-syn aggregates is limited by the level of endogenous α-syn available in the cell, and that the aggregation process is amplified in the presence of elevated levels of monomeric α-syn [15–17]. Studies performed in rats [11, 13, 18] confirm that this is the case also in nigral DA neurons *in vivo*. Thus, the use of this combined approach makes it possible to speed up the pathogenetic process and induce more prominent DA neuron loss and motor impairments, accompanied by extensive α-syn pathology and a prominent inflammatory response akin to what is observed in patients [13, 18, 19]. In this combined AAV-α-syn/fibril (SynFib) model, the AAV-α-syn vector and the PFFs are injected unilaterally into the SN at doses that have only moderate impact when each component is administered individually. Importantly, the AAV-α-syn vector is used at a lower dose, compatible with a more physiological level of α-syn expression and closer to that seen in PD patients.

The purpose of this article is to summarize the experience gained so far from the use of the unilateral SynFib model in rats and mice, and discuss important parameters of the model that affects its performance, to be used as a guide for others to apply this method in their research.

## COMMENTS ON THE METHOD

### AAV vector

The purpose of the vector is to increase the α-syn level in the DA neurons in order to amplify the generation of toxic α-syn aggregates from the fibril seeds. Ideally, the induced α-syn level should be kept within the range seen in PD patients, i.e., up to 2-3-fold above normal. Since the *in vivo* efficiency of individual AAV-α-syn batches varies greatly due to a number of factors, including the AAV serotype, the promoter and enhancers used in the vector construct, the production and purification method, and the method used for assessment of the genome copy (gc) number, the optimal working dose cannot be determined by the gc titer alone. The importance of not depending on the gc titer is further underscored by the fact that the gc titer is highly unreliable as predictor of the infectious particle titer, i.e., the *in vivo* efficiency of the vector. For each new vector batch, therefore, we recommend selecting the working dose on basis of the spread and toxic impact of the vector-derived α-syn in a pre-test experiment in rats. In this pre-test we inject the vector alone at a single site, and the extent and impact of vector-derived α-syn is determined at 3–4 weeks post-injection using antibodies against TH and human α-syn.

**Fig. 1:**
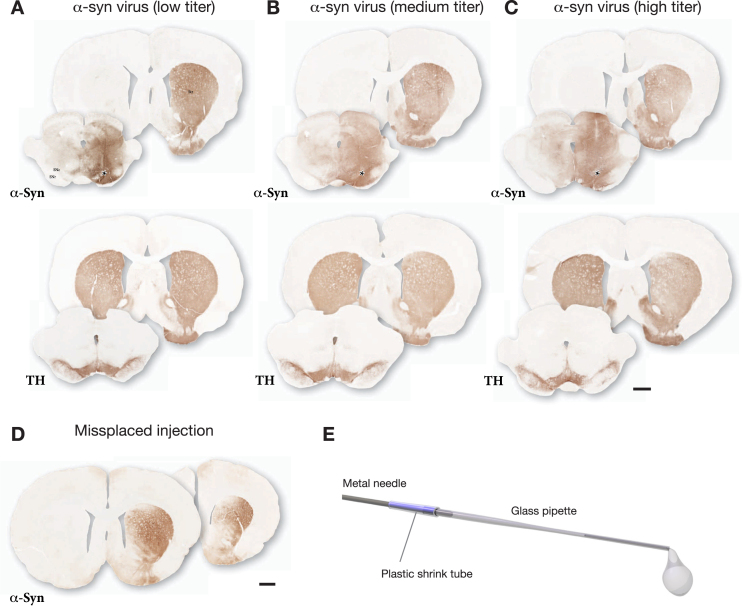
Example of the outcome of a pre-test made in order to select a suitable dose for the AAV-α-syn vector. The vector was injected at 3 different doses as a single 3μL deposit in the SN and the brains were processed for immunohistochemistry three weeks later. The extent and distribution of vector-derived α-syn was determined in sections through striatum and SN using antibodies against TH (Sheep ab1542) and human α-syn (Rabbit ab5038 from Millipore). The selected low titer dose illustrated in A showed good spread of AAV-derived human α-syn, covering the medio-lateral extent of the SN, and α-Syn+terminals (expressing α-syn as a result of anterograde transport) was seen to cover the entire medio-lateral extent of the caudate-putamen. This wide-spread distribution of α-syn-expressing terminals indicates that the vast majority of nigral DA neurons (as well as some VTA neurons) had been transduced by the vector. TH immunostaining showed no clearly detectable TH+cell loss, or loss of TH+innervation in the striatum. The medium titer dose illustrated in B (5-fold higher than in A) showed some spread of α-syn into the contralateral side and reduced TH staining in both striatum and SN. At the highest dose illustrated in C (16.5-fold higher than in A) the vector-derived α-syn extended well beyond the ventral midbrain/SN area and spread also across the midline into the contralateral non-injected side, and there was a clear loss of TH+neurons in the SN and TH+innervation in the striatum. For this vector batch the selected dose was 3.6 × 10^9^ gc in 3μL (titer determined by PCR using a probe recognizing the ITR). D shows the uneven expression of α-syn in striatum resulting from a minor displacement of the injection, 0.7 mm lateral to the one shown in A–C. E shows the glass capillary, attached to the 22-gauge metal cannula of a Hamilton syringe, used for the injections. Asterisks mark the injection sites. Scale bars in A–D: 1 mm.

The outcome of one such pre-test is illustrated in Fig. 1. The vector was injected at 3 titers (*n* = 3 rats/titer) and the impact of the vector was analyzed after 3 weeks. In this case the lowest titer (diluted to 2% of the stock solution; Fig. 1A) was selected for use based on the following features: i) The vector-derived α-syn covered the entire mediolateral extent of SN, and α-syn immunostaining was evenly spread over the entire medio-lateral extent of the striatum; ii) TH immunostaining showed no clearly detectable cell loss, or loss of TH+innervation in the striatum. At the medium dose (diluted to 10% of the stock solution; Fig. 1B) there was some spread of α-syn into the contralateral side and reduced TH staining in both striatum and SN. At the highest dose (diluted to 33% of the stock solution; Fig. 1 C) the vector-derived α-syn extended well beyond the ventral midbrain/SN area and spread also across the midline into the contralateral non-injected side, and there was a clear loss of TH+neurons in the SN and loss of TH+innervation in the striatum. It should be noted that at this early timepoint the loss of TH+cells and fibers is primarily due to downregulation of TH rather than cell death [20]. Based on this comparison, the low dose was judged as optimal for the combination with PFFs. For this vector batch the selected dose was 3.6×10^9^ gc in 3μL (titer determined by PCR using a probe recognizing the ITR).

In our experiments in rats and mice, we have used a vector of the AAV 2/6 serotype, with human wt α-syn driven by the human synapsin-1 promoter, as described [21], but other AAV serotypes, such as AAV 2/1, 2/5, 2/7, 2/8, and 2/9, and other promoters, such as the CBA and PGK promoters, are equally effective in inducing α-syn production in nigral DA neurons (see [3] for review).

### Pre-formed α-syn fibrils (PFFs)

The PFFs used in our experiments have been prepared in Kelvin Luk’s lab from full-length recombinant human α-syn and sonicated before use according to the method described by Volpicelli-Daley et al. [22]. The PFFs were used at a concentration of 5μg/μL. Since the aggregation process is more efficient if the PFFs and the endogenous α-syn are from the same species [23], we have opted to use human PFFs to trigger aggregate formation from human α-syn, expressed by the AAV vector, thus providing a “humanized” model of α-syn aggregate formation. The need for high quality and standardized materials and protocols for PFF generation is well recognized and both mouse and human α-syn monomers formulated to generate standardized PFFs are now available via a collaboration between Michael J. Fox Foundation and Proteos (https://www.proteos.com/partnerships/mj-fox-partnership).

### Stereotaxic injections

The AAV-α-syn vector and the sonicated human wt PFFs (5μg/μL) are mixed 1 : 1, immediately before use, and injected as follows:

*In Sprague-Dawley (SD) rats (Charles River):* Four μL of the mixed preparation (or AAV-α-syn or PFFs alone) is injected unilaterally into the substantia nigra (SN) at two sites (2μL/site): 1) A/P -5.3 (from bregma), M/L -1.6, D/V -7.2 (from dura) and 2) A/P -5.3, M/L -2.6, D/V-6.7, with the head in “flat scull” position, i.e., with bregma and lambda at the same level plane.

The use of two injections is recommended in order to increase the spread of the PFFs and the vector and reach as many of the nigral DA neurons as possible. Two injections are also advantageous since it makes the outcome less sensitive to variation in the exact placement of the injection which can be difficult given the small size of the SN. Figure 1D shows an example that illustrates this point. In this animal a single injection site was used, as in Fig. 1A, but the injection was placed 0.7 mm lateral to the one illustrated in A. As a result, the vector failed to target the medial part of the SN and the α-syn staining was limited to the central-lateral parts of the striatum. Misplacement of this magnitude has less impact when the injected volume is spread over two injection sites.

The injections are made at a rate of 0.2μL/min using a 10μL Hamilton syringe equipped with a 22-gauge needle (0.7 mm outer diameter). In order to minimize the damage caused by the injection the needle is fitted with a thin glass capillary drawn to a tip with 250μm (outer diameter) opening (Fig. 1E). To prepare the drawn glass capillary (Sutter borosilicate B150-110-10), we use a Sutter Instruments pipette puller (model P-1000) equipped with a specially ordered 10 mm coil. A small (1μL) air bubble is drawn into the capillary, followed by the volume used for the injection. Observing the movement of the bubble help to ensure that the entire volume is delivered. After infusion of the entire volume, the needle is left in place for an additional 2 min before being slowly removed to minimize back-flow.

As an alternative to this pipette-fitted devise the Baekelandt lab has opted to use a 10μL Hamilton syringe equipped with a 30-gauge needle (0.3 mm outer diameter) for their injections [11]. Although the 22-gauge needle is stiffer and thus less prone to bending, fine results have been obtained also with the thinner injection needle, and the amount of non-specific damage appears to be similar with both devises.

*In nude athymic rats (Envigo):* We have transferred the model to athymic nude rats in order to allow studies of human-to-rat xenografts in the absence of immunosuppression. The AAV/PFF mixture (1 : 1) is injected at two sites in the SN, as in SD rats, above, but since the brain in nude rats is smaller in size the coordinates need to be modified to: 1) A/P -5.0, M/L -1.6, D/V-6.8; and 2) A/P -5.0, M/L -2.6, D/V-6.1 (flat scull position).

*In C57BL/6 mice:* The AAV/PFF delivery was applied in two versions: either as a single injection of the 1 : 1 AAV/PFF mixture, or as two separate injections made four weeks apart, using the same injection devise as used in rats (Fig. 1E). *The mixed AAV/PFF injection* was made unilaterally as a single 2μL deposit in the SN at the coordinates: A/P -2,8 (from bregma), M/L -1.1, D/V -4.3 (from dura) (flat scull position). The injection was made at a speed of 0.2μL/min and the needle was left in place for an additional 2 min to minimize back flow. As for the rats, above, a small air bubble is drawn into the capillary, followed by the volume used for injection. Observing the movement of the bubble ensures that the entire volume is delivered. *In the alternative staggered version*, the delivery was done in two steps: In the first step a single 1.5μL of the AAV-α-syn vector was followed four weeks later by an injection of 2μL PFFs, injected into the same site and using the same syringe and injection speed as above.

## DA NEURON CELL LOSS

**Fig. 2:**
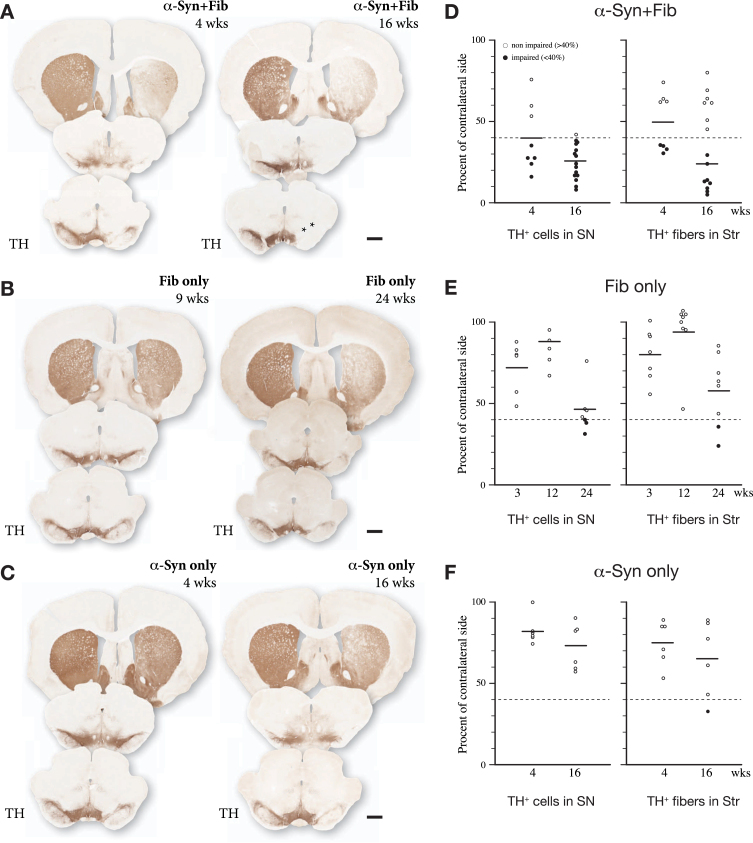
Toxic impact of a mixed AAV/PFF injection into the SN (A, D), compared to the effect of PFFs alone (B, E), or the AAV-α-syn vector alone (C, F), as assessed at 4- and 16–24 weeks post-injection. In the AAV/PFF animals a significant TH+cell loss,>60%, was seen in most of the rats already at 4 weeks, and in over 90% at 16 weeks. In the rats injected with PFFs alone cell loss of this magnitude was obtained only at the longest time point (24 weeks), and this was seen in only one-third of the PFF-injected rats at this timepoint. At the moderate dose used here, the TH+cell loss induced by the AAV-α-syn vector alone was overall less than 50%, even at the longest timepoint (16 weeks). Scale bars in A–C: 1 mm. Data compiled from refs [13] and [18].

As summarized in Fig. 2, the extent of TH+cell loss and the loss of TH-innervation in the striatum is much more pronounced in rats receiving the combined AAV/PFF injection (A, D) compared to the animals receiving the PFFs alone (B, E) or the AAV-α-syn vector alone (C, F) administered in the same doses as used in the mixed injections. In the AAV/PFF treated animals, TH+cell loss of a magnitude compatible with the development of measurable motor impairments (>60% nigral cell loss; see [5, 24]) was observed in about 60% of the animals already at 4 weeks post-injection and had developed in all rats, but one, at 16 weeks (Fig. 2D). At this time point the TH+cell loss varied between 58 and 92%. In animals receiving the PFFs alone, TH+cell loss of similar magnitude developed only at the longest time point (24 weeks), and then only in a less than half of the rats (Fig 2E). The cell loss seen in the AAV only group was overall less than 50%, even at the longest timepoint (16 weeks) illustrated here (Fig. 2F).

## 3 IMPACT ON SPONTANEOUS AND DRUG-INDUCED BEHAVIOR

**Fig. 3:**
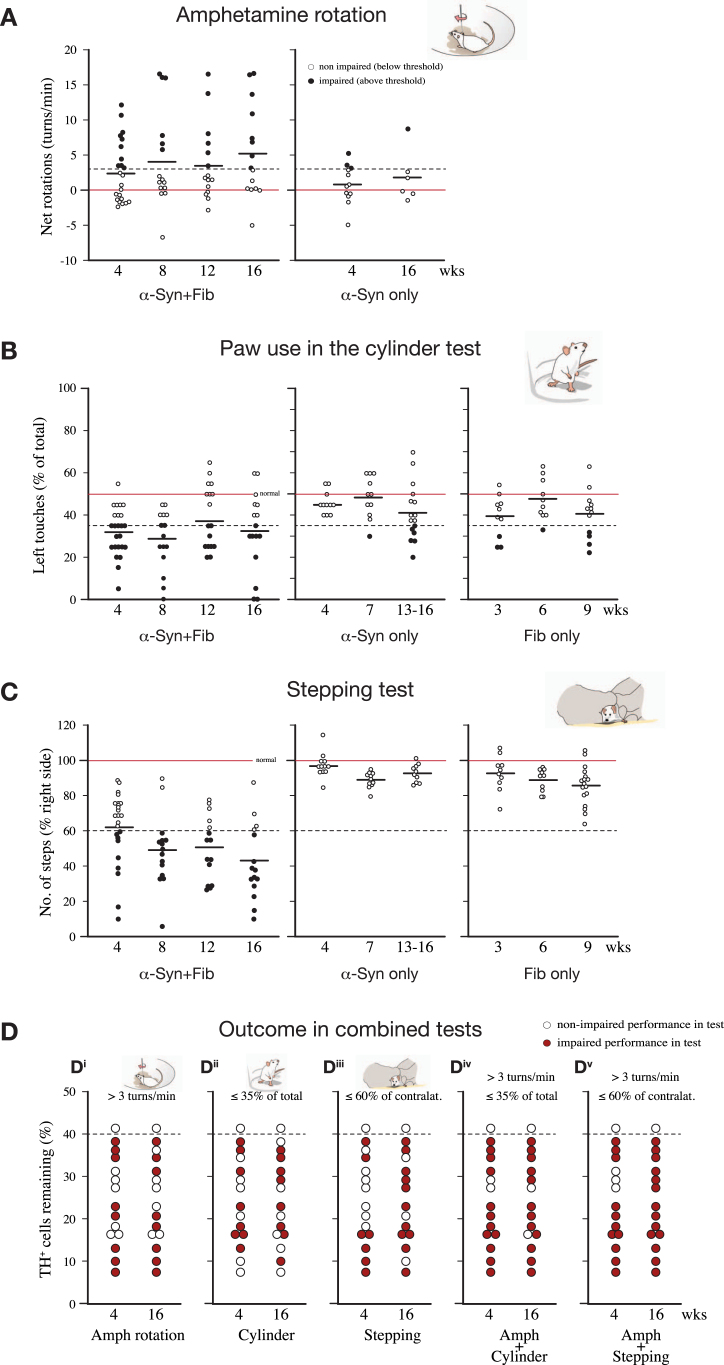
The combined AAV/PFF injection induces more profound motor impairments than AAV-α-syn or PFFs alone, as observed in the amphetamine rotation (A), cylinder (B), and stepping (C) tests. In the experiments summarized here (compiled from [13] and [18], combined with unpublished data), the AAV/PFF treated rats developed significant impairments in all three tests already at 4 weeks post-op, and this level of impairment was maintained, or increased, at longer timepoints. In the PFF only and AAV-α-syn only groups the impairment did not exceed the limits for significant functional impairment in either the amphetamine test (set at > 3 turns/min) or in the stepping test (60% of intact side), even at the longest timepoints. In panel D, the plots give the magnitude of TH+cell loss in the individual rats in the mixed AAV/PFF group. The red dots indicate rats showing significant impairment in either of the three tests (panel D^i^-D^iii^), or impairments in the rotation test and/or cylinder tests combined (panel D^iv^), or the rotation and/or stepping tests combined (panel D^v^). As discussed in the text, this points to the possibility to use scores from a combination of two tests as a screening tool to predict the magnitude of TH+cell loss that will develop long-term in the AAV/PFF treated animals.

As a result of increased and accelerated cell loss, impairment in three standard motor tests–amphetamine-induced turning behavior in the rotation test, paw use in the cylinder and stepping tests, and–is more pronounced and appears earlier in the animals receiving the combined AAV/PFF injections than in those receiving AAV-α-syn or PFFs alone. In the experiment summarized in Fig. 3, significant impairment in the AAV/PFF treated animals was evident in all three tests already at 4 weeks post-injection, and was maintained, or further increased, at later timepoints. The impairment seen in the Fib only and α-syn only animals, by contrast, was of lesser magnitude and, with single exceptions, did not exceed the limits for a functionally significant impairment in any of the three tests, here set at > 3 ipsilateral turns/min in the amphetamine rotation test (Fig. 3A); 35% of total touches with the contralateral paw in the cylinder test (Fig. 3B); and 60% of intact side in the stepping test (Fig. 3C).

On an individual rat-to-rat basis the magnitude of impairment does not correlate well with the magnitude of TH+cell loss seen in each animal. Nevertheless, the performance in the three tests can be quite informative. In the experiment summarized in Fig. 3D, 14 of the 15 AAV/PFF treated rats had a TH+nigral cell loss of more than 60% (as assessed long-term, see Fig. 2D). Between one-half and two-thirds of these well-lesioned rats showed impaired performance in the individual tests (7-9 rats, but not always the same rats, passed the criterion in each of the three tests, marked by red circles in Fig. 3D), and the outcome was similar at both timepoints, 4- and 16-weeks post-injection. Thus, a single test is not sufficiently reliable to assess extent of impairment. Combining the results of the rotation test with either the cylinder or stepping tests, however, made it possible to correctly identify between 80 and 100% of the severely lesioned animals. As illustrated in the two right-hand panels in Fig 3D, the rats marked by red dots passed the criterion in either one of the tests, or in both tests, and all of them had a TH+nigral cell loss > 60% (assessed at 16 weeks). Interestingly, this correlation was seen also when the tests were performed early, at 4 weeks. At this early timepoint, 11–12 of the 14 well-lesioned rats fulfilled this combined criterion. This suggests the possibility to use a combined criterion of *either* > 3 turns/min in the rotation test *or* 35% of total touches in the cylinder test (alternatively 60% of intact side in the stepping test) to distinguish rats with > 60% nigral cell loss form those with less severe lesions. Our data suggest that this combined criterion can be applied as a screening tool already at 4 weeks and used as a predictor of the magnitude of nigral damage that will develop long-term, in this case at 16 weeks.

## INDUCTION OF α-SYN PATHOLOGY AND INFLAMMATORY RESPONSE

**Fig. 4:**
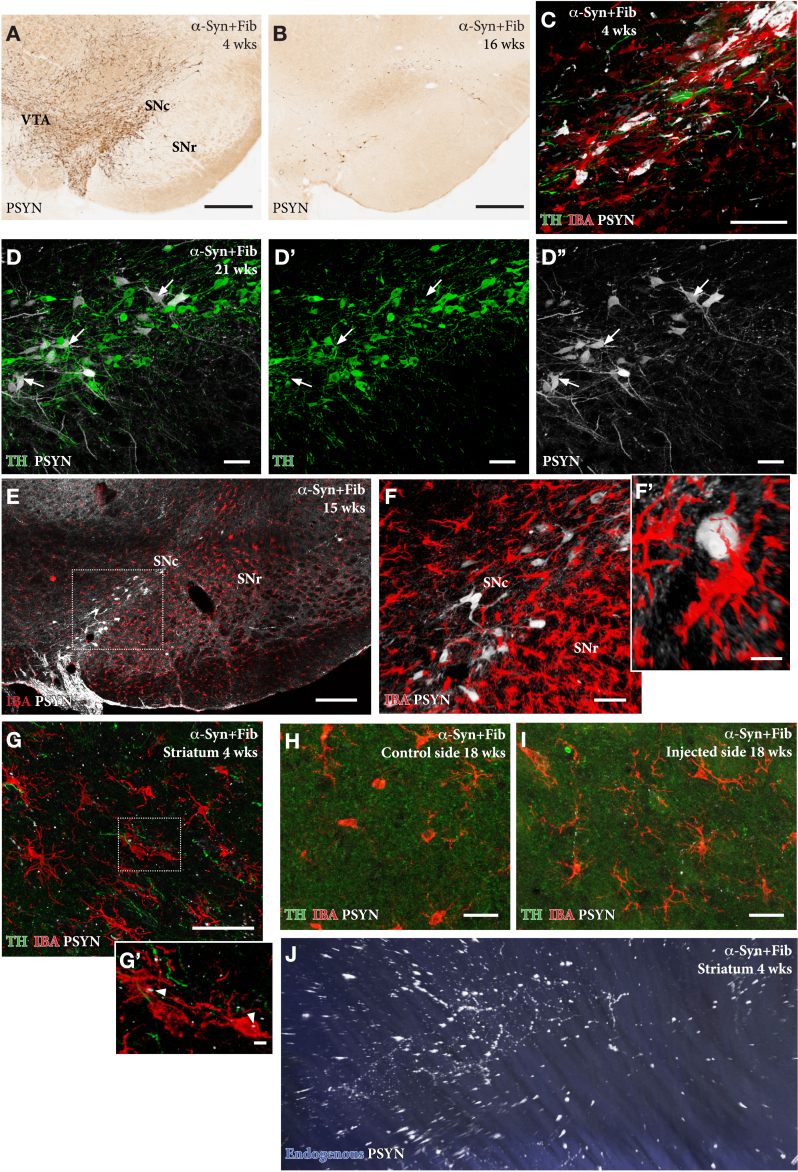
In the AAV/PFF treated rats prominent p-syn+pathology develops early, already at 4 weeks, in the DA neurons (A, C), as well as in their axons and terminals in the striatum, as illustrated in the Supplementary 3D movie captured by light-sheet microscopy (Supplementary Movie S1; still image in J). This is accompanied by an Iba1 + microglial response (F, G, H) and downregulation of TH (arrows in D-D''), that is maintained also at longer time-points. The p-syn pathology declines over time (B, E, F) which is in line with the progressive loss of the affected DA neurons. A notable feature of the microglial response is the appearance of p-syn+inclusions inside the Iba1 + microglia (arrow heads in G'). Scale bars: 500μm (A,B), 50μm (C,D,F,G,H,I), 200μm (E), 10μm (F',G').

A distinguishing feature of the SynFib model is the early development of inclusions and aggregates positive for phospho-Ser129-α-syn (p-syn) in the affected nigral DA neurons (Fig. 4A–F), as well as in distorted axons and terminals distributed along the nigrostriatal pathway and within striatum (Fig. 4G–J; see Supplementary 3D movie S1 of axonal pathology in the striatum, panel J) [13, 18, 19]. The p-syn+pathology seen in the SynFib model is much more prominent, develops earlier, and involves larger numbers of nigral neurons than is the case in either the AAV only or PFF only models. Similar to the Lewy bodies seen in human PD these inclusions are ubiquitin positive and resistant to proteinase-K digestion [18]. In the AAV only model the appearance of the p-syn+pathology is different in that it is diffusely distributed in the cytoplasm and often combined with a prominent localization to the nucleus [13].

In the SynFib model, the emerging pathology is seen already at 4-weeks post-injection and is accompanied by a prominent microglial response (Fig. 4 C) and downregulation of TH in most, but not all, p-syn+nigral neurons (arrows in Fig. 4D) [13, 18]. The activated Iba1 + microglia are confined to areas of ongoing pathology and are seen to surround the p-syn+neurons and processes. p-Syn immunostained inclusions occur also inside Iba1 + microglia (arrows in Fig. 4G'), indicating cell-to-cell transfer of seeding material or active scavenging of cellular debris. As described by Thakur et al. [13] and Hoban et al. [19], the p-syn+nigrostriatal pathology and the magnitude of the microglial response decline over time, which is in line with the progressive degeneration of the affected nigral neurons. However, some surviving p-syn+neurons, as well as a moderate microglial response, remain over long time (Fig. 4F, H) at a level exceeding that seen in the AAV only model at this moderate titer of the vector [13].

## APPLICATION OF THE SynFib MODEL IN NUDE ATHYMIC RATS

In order to confirm that the SynFib model can be applied also to nude (Envigo) rats we performed an experiment where AAV-α-syn and PFFs were co-injected unilaterally at two sites in the SN, as in SD rats above, and the brains were analyzed at 18- and 24-weeks post-surgery. Since the brain in nude rats is smaller in size we found that the coordinates needed to be modified, using the coordinated given above. Similar to the observations made in SD rats we detected a substantial loss of TH+neurons on the injected side, accompanied by extensive expression of p-syn in the surviving nigral TH+neurons and induction of activated Iba1 + microglia in the area of p-syn+pathology. As in SD rats p-syn+inclusions were observed in some of the activated Iba+cells in the SN. At the level of the striatum, we observed a wide-spread loss of TH+fibers, and p-syn+aggregates in fibers and terminals as well as larger p-syn+inclusions in swollen and distorted pre-terminal axons scattered throughout the striatum. None of these pathologies were found on the intact contralateral side.

## APPLICATION OF THE SynFib MODEL IN MICE

Although the SynFib model was initially developed for use in rats, i.e., in a species that allows for more consistent functional readouts in behavioral tests, and thus serve as a complement to the standard unilateral 6-OHDA rat model, we have performed an exploratory experiment to find out whether combined AAV/PFF delivery can work also in mice. AAV-α-syn and PFFs were either delivered together in a single intranigral injection (*mixed injection*), or the PFFs were delivered 4 weeks after the AAV-α-syn injection (*delayed injection*), and the mice were killed 12 weeks later. The injections were made at a single site into the right SN. In this experiment we used two tests, apomorphine rotation and paw use in the cylinder test, that have been found to work well in mice [25].

**Fig. 5:**
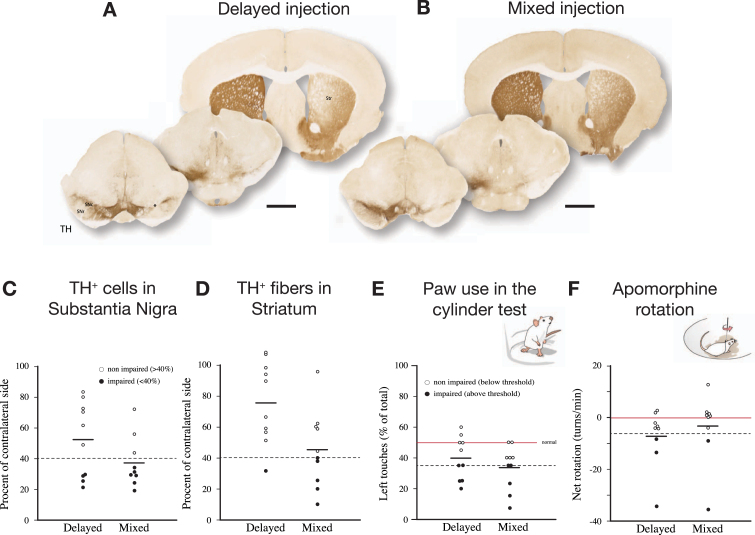
The SynFib model is also applicable in mice. In the experiment illustrated here PFFs were injected together with AAV-α-syn (“Mixed”) or 4 weeks after the AAV injection (“Delayed”). The injections were made at a single site in the SN, unilaterally, and the mice were analyzed 12 weeks later. The extent of TH+cell loss and behavioral impairment induced by the mixed AAV/PFF injection was similar to those seen in rats (see Fig. 2), and they tended overall to be more pronounced than in the delayed group. In the rotation test one mouse of each group did not move and were for this reason not included. In one mouse of the Mixed group cell counting could not be performed due to damage caused during sectioning. Scale bars in A and B: 1 mm. Unpublished data.

**Fig. 6:**
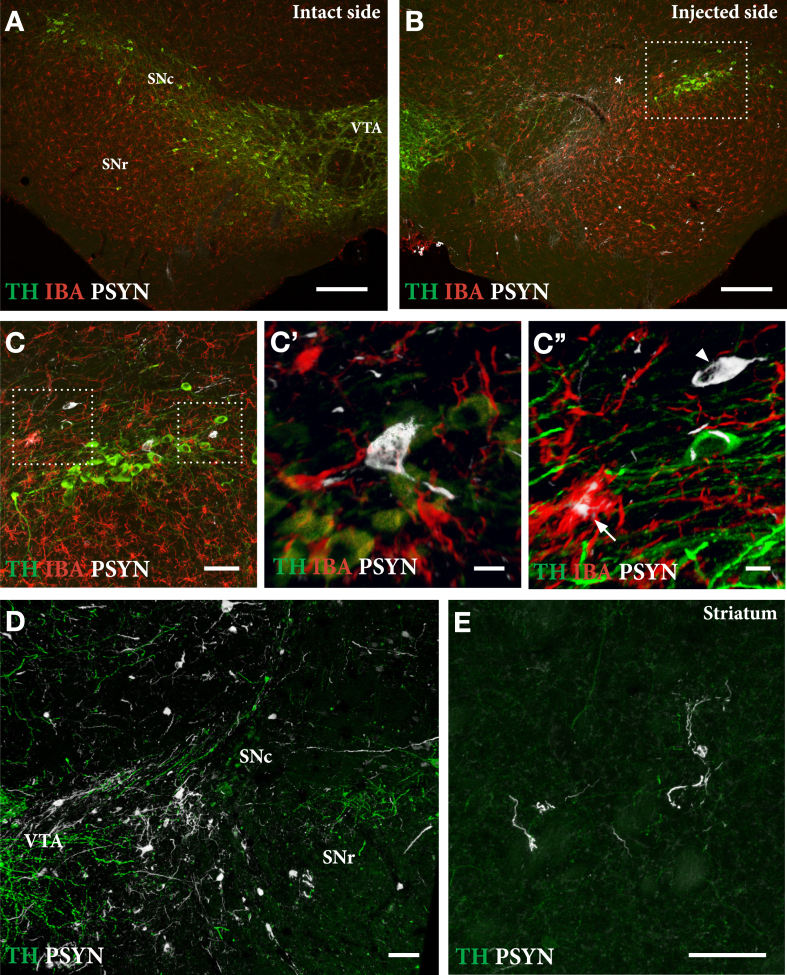
Extent of TH+nigral cell loss (A, B) and p-syn+pathology seen in nigral DA neurons (C, D) and striatal terminals (E) in mice treated with a single intranigral injection of AAV/PFFs. The framed square in B is shown at higher magnification in C, and the squares in C are shown at higher magnification in C' and C''. At this stage, 12 weeks post-injection, the ongoing p-syn pathology and microglial response is similar in magnitude and appearance to that seen in long-term AAV/PFF treated rats (see Fig. 4). As in rats, TH downregulation (arrowhead in C'') and p-syn+inclusions inside activated Iba1 + microglia (arrow in C'') occur also in the mouse SynFib model. Scale bars: 200μm (A,B), 50μm (C,D,E), 10μm (C',C''). Unpublished data.

As shown in Fig. 5, delivery of PFFs mixed with the AAV vector tended overall to be more efficient than when the PFFs were given with a 4-week delay. In the mixed group, 6 of the 9 mice showed a TH+nigral cell loss greater than 60% (Fig. 5 C), and 5 of them were also impaired in the paw use test (35% of total touches with the contralateral paw; Fig. 5E). In the apomorphine rotation test only two displayed a significant turning rate,>3 turns/min, indicating that the rotation test was less informative than the cylinder test in detecting TH+cell loss in AAV/PFF treated mice. The α-syn pathology and associated microglial response, as revealed in the p-syn and Iba1 stained sections from the nigra (Fig. 6A–D) and striatum (Fig. 6E), seen at this long term timepoint (12 weeks), was similar in magnitude and appearance to that seen in the long term AAV/PFF treated rats, as described above. As in rats, downregulation of TH (arrowhead in C''), and p-syn^+^ inclusions inside activated Iba1^+^ microglia (arrow in C''), occurred also in the AAV/PFF treated mice.

## DISCUSSION

In the SynFib model, the delivery of α-syn PFFs together with the AAV-α-syn vector offers several advantages over the standard PFF model due to the enhanced and accelerated α-syn pathology induced by the PFF seeds in the presence of an elevated, but physiologically relevant, α-syn level. Significant cell loss and behavioral impairments are observed already at 4 weeks, and they are more extensive compared to the slow degenerative changes that take around 6 months to appear when the PFFs are administered alone [8–11]. In the experiments conducted so far, the long-term nigral TH+cell loss has been consistently above 50% provided that the injections have been correctly placed. Similar levels of nigral cell loss can be obtained in the AAV-α-syn only model but only at very high α-syn expression levels [5–7]. Thus, compared to AAV-α-syn alone, the SynFib model is advantageous in that the α-syn expression level induced by the AAV vector is kept at a moderate and more physiological level, and that the addition of PFF seeds facilitates the formation of α-syn aggregates, accompanied by a more pronounced inflammatory/microglial response.

The SynFib model shares features with the 6-OHDA lesion model in that it is applied unilaterally in a single dose, and that the toxic impact is sufficient to induce significant impairments in both spontaneous and drug-induced motor behavior in a high percentage of the treated animals. In contrast to the 6-OHDA lesion model, however, which is essentially non-progressive and lack α-syn related pathology and inflammatory response characteristic of human PD, the damage to the nigrostriatal DA neurons seen in the SynFib model is progressive and accompanied by the development of α-syn pathology and a time-dependent microglial activation. Although the magnitude of cell loss seen in the SynFib model is less than that obtained with 6-OHDA injections into the medial forebrain bundle (MFB), it is quite similar to that seen in rats with intrastriatal 6-OHDA lesions [2, 26].

In the experiment summarized in Figs. 2 and 3, the nigral TH+cell loss obtained long-term varied from 58 to 92%, and significant impairment in drug-induced and spontaneous motor behavior is observed already at 4 weeks in 1/2 to 2/3 of the injected animals. Similar variability is seen in groups of rats with 6-OHDA injections in striatum or MFB, although, in this case, amphetamine-induced rotation can be applied as a pre-test to exclude rats with incomplete lesions. In both models, the rotational or paw-use scores do not correlate well with the extent of TH+cell loss on the individual rat-to-rat level [2]. This mismatch is readily explained by differences in the extent and distribution of the denervated areas in the striatum. Striatum is functionally heterogenous, and different sectors have been shown to subserve different aspects of motor function. Turning behavior, for example, is driven by the DA projection innervating the dorso-lateral sector of the striatum. It is likely, therefore that the amphetamine-induced turning rate is determined by the extent of sparing of the DA innervation within this subregion [27], a factor that will inevitably vary from animal to animal independent of the overall magnitude of nigral cell loss and striatal denervation. Nevertheless, our data suggest that the amphetamine rotation score, in combination with the scores obtained in either the cylinder or the stepping tests, may be used as a predictor of the long-term loss of nigral TH+neurons and the accompanying loss of TH+innervation in the striatum. Thus, a combined criterion of *either* > 3 turns/min in the amphetamine test *or* 35% of total touches in the cylinder test (alternatively 60% of intact side in the stepping test), recorded at 4 weeks, can be used to identify rats with more than 60% loss of nigral TH+neurons, as recorded at longer timepoints.

Importantly, the impairments seen in the α-syn models result from a combination of cell death and dysfunction of remaining DA neurons, which is in contrast to the toxin models where the behavioral deficits that are caused by the acute degeneration of DA neurons and their terminals. In the α-syn overexpression or fibril models, the degenerative changes have been shown to start in axons and terminals [5, 20, 28, 29], and at early stages the loss of TH-positive neurons in the SN is in part (in our experiments around 50%) due to downregulation of TH in p-syn containing, dysfunctional DA neurons [9, 10, 30]. The affected p-syn containing DA neurons targeted by the vector and PFFs are gradually lost over time, but even at longer time-points part of the nigral neurons and their axons remain with their load of p-syn positive aggregates and inclusions, indicative of an ongoing degenerative process. These changes make it possible to distinguish two phases in the degenerative process seen in the SynFib model: *An early phase* that develops over the first month, characterized by the appearance of p-syn-positive inclusions that involve not only the cell bodies, but also the dendrites, axons and axon terminals of the nigral DA neurons (see Supplementary Movie S1), accompanied by downregulation of TH and other phenotypic markers and a prominent immune/inflammatory response, followed by *a second phase* characterized by a progressive loss of the affected DA neurons that takes place over the following weeks and months. This stepwise progression of the degenerative process provides a useful time-window for disease modifying interventions in the SynFib model.

## CONCLUSIONS

• The SynFib model combines the key features of the α-syn-PFF and AAV-α-syn models, i.e., the generation of toxic p-syn positive aggregates and Lewy body like inclusions induced by the fibril seeds, in combination with the increased vulnerability caused by increased cellular levels of α-syn (driven by the AAV vector).
• The induction of Lewy-like p-syn pathology in nigral DA neurons and processes is both accelerated and enhanced and is prominent already within the first month.
• The degenerative changes are progressive, characterized by an early phase of p-syn aggregation, axonopathy, and downregulation of the DA phenotype, accompanied by microglial activation and followed by a progressive loss of the affected DA neurons that takes place over the following weeks and months.
• Injection of the AAV/PFF mixture into the SN makes it possible to target a large proportion of the nigral DA neurons and obtain a level of DA neuron cell loss (>60%) needed to induce impairments in drug-induced and spontaneous motor behaviors.
• The scores obtained in the amphetamine rotation test, in combination with those obtained in the cylinder or stepping tests, applied at 4 weeks post-injection, can be used as a selection criterion to identify rats with a functionally significant TH+cell loss (>60%).

The SynFib model shares attractive features of the standard 6-OHDA lesion model: a single unilateral stereotaxic intervention; pathology and cell loss developing over a short time span; and the possibility to monitor the degenerative changes using tests of motor behavior. The two methods are clearly complementary in that they replicate different aspects of the pathogenesis of human PD: ROS-dependent neurodegeneration in the 6-OHDA model, and α-syn-induced progressive pathology and neuroinflammation in the SynFib model. The 6-OHDA model will remain the model of choice for studies requiring near-complete removal of the midbrain DA system and profound DA-related motor impairments, while the SynFib model provides a more disease-relevant model for studies of α-syn-related pathogenesis and disease modifying treatments. The SynFib model, moreover, is attractive as a routine experimental tool in that it combines the attractive features of the commonly used AAV and PFF models, i.e., the generation of Lewy-like aggregate pathology characteristic of the PFF model and the speed of development of α-syn pathology and cell death seen in the AAV model, in combination with more prominent and long-lasting inflammatory response.

## CONFLICT OF INTEREST

The authors have no conflict of interest related to the work discussed in this review.
